# Merycism

**Published:** 1897-06

**Authors:** Lewis S. Somers

**Affiliations:** Philadelphia, Pa.


### MERYCISM.

By LEWIS S. SOMERS, M. D., PHILADELPHIA, PA.

The voluntary act of regurgitating the food for the purpose of remastication is the normal method of digestion in a large class of the lower animals. To this condition the name merycism or rumination has been applied, the latter term being used most frequently and often wrongly, the condition being erroneously associated with involuntary regurgitation. Occurring occasionally in the human subject, it is most frequently seen in degenerates or neurasthenics, or as the result of prolonged mental strain, and usually bearing a remarkable parallelism to the degree of the neurasthenia. Nacke has reported his own case; at the age of thirty years, becoming neurasthenic from excessive mental work, he was subject to periodical attacks of merycism.

The mechanism of the condition starts with the act of regurgitation, which is preceeded by a rapid inspiratory effort and simultaneous closure of the glottis, during which the diaphragm is forced downward, with contraction of the stomach walls, so drawing the contents of the stomach through the cardiac orifice; this is immediately followed by a deep expiration to prevent the return of the food. The stomach contents are regurgitated to the mouth in small quantities, then masticated and again swallowed, the process being repeated at intervals until complete digestion has ensued or another meal been partaken.

The following subject, presenting the various phenomena of merycism, applied to the aural clinic of the Union Mission Hospital, October 11, 1893, for relief from progressive impairment of hearing:

Albert McC——, male, aged five years. Family history is negative, except as to an uncle and cousin on the maternal side, neither of whom could talk until after their sixth year.

Personal history: Born at term, nearly asphyxiated on account of prolonged labor; with this exception delivery was normal, no instruments being used. Attention at this time was attracted to the large size of his head. He was unusually intelligent as an infant, and with the exception of an indefinite history of coryza and convulsions at three months of age, was healthy until his third year, when he had measles. Previous to this he could talk as well as any child of the same age, and hearing was normal, but the rubeola was followed by a total loss of hearing without aural suppuration, and in a short time he could not talk, simply making unintelligible sounds. He has one brother, ten years of age, who is healthy. He has been well, as regards general health, since the attack of measles.

The boy was robust in appearance, could not talk, and presented the appearance of chronic hydrocephalus as far as the size of his head was concerned. Measurements of the head were made, and showed a marked increase in size over the normal. The hard palate was "gothic" shaped, there was slight hypertrophy of the tonsils, and the pharynx was sclerotic. Nose, turbinates hypertrophied. Ears, both membrana tympani thickened and retracted. He could not hear watch or voice on contact, but bone conduction was normal. Under treatment hearing was restored, and he could talk fairly well.

He was then absent from the clinic for about six months, when his mother brought him back, with the complaint that "he ate his food like a cow." On questioning, it was ascertained that he would regurgitate his food and masticate it with apparently greater enjoyment than during the original eating. This was entirely voluntary on his part, and occurred at irregular intervals, but principally about one-quarter to one-half hour after meals. Frequently he would hide to avoid censure, and when found would be sitting alone, masticating the remains of a former meal with evident relish. When this condition was first noticed, true vomiting would sometimes take place from his efforts to regurgitate more than his mouth would hold; but this disappeared as his experience increased, for he soon became able to judge of the amount required to masticate at one time. Dr. John H. Dripps then saw the case with me, and made a diagnosis of neurasthenia. The patient was put on a semi-solid diet, meat in small pieces to be eaten slowly, and the mother instructed carefully to attend to his meals and use large amounts of "moral suasion." He also received the syrup of the iodide of iron and calisaya. Improvement was gradual, but at times he would apparently relapse into the former condition of merycism. When last seen, about six months ago, his mother stated that he remasticated his food only at long intervals, and she considered him cured.

A lowered or perverted condition of the nervous apparatus plays an important part in the etiology of merycism. Heredity is of some importance, as concerned in the transmission of a vitiated nerve tone. This hereditary influence was in the minds of some of the earlier writers when they laid stress on the descent of the person affected with the disorder from herbivorous parents, or thought their ancestors possessed two stomachs, like some of the lower animals. In a number of cases studied by different authors, neurasthenia was credited with being the underlying condition, and at the same time some disorder of the stomach was present. Lemoine and Linassier divide the condition into two varieties, a simple and a pathological form, while they have divided the latter form into several sub-varieties depending upon the degree of dyspepsia present. Mimicry has appeared to be a factor in a few reported cases. A low grade of stomach irritation probably present in nearly all cases, and by reflex action gives rise to the desire to regurgitate the food; this irritation may be the result of local pathological changes or simply a manifestation of lowered nerve tone.

The frequency with which merycism is seen among the insane, and the fact that it may be voluntarily produced or even suppressed, are in favor of its nervous etiology. Improper mastication of the food probably plays an important part in its etiology. Hammond describes a case in which rapid eating was productive of the condition, it being usually absent when the food was taken slowly and properly masticated. In these cases it appears to be an effort of nature to secure sufficient mastication, as when the food is regurgitated proper mastication always occurs, from the pleasure afforded the patient both by the mere act of chewing the food and by the pleasant taste associated with it.

A study of the regurgitated food has shown that digestion had not commenced or occurred to any great extent, thereby not interfering with the pleasant taste which seems to be excited. Analysis of the stomach juices has been productive of so widely varying and inconsistent results that no definite changes have been observed; in not a few cases the chemical examination showed nothing differing from the normal.

Merycism takes place usually on an average of fifteen minutes after a full meal, but the time may be delayed to over an hour and varies with the individual case, depending upon the size of the repast, or the haste in which it was partaken, or whether severe exercise occurred immediately after the meal. The time consumed in the second is usually greater than during the original mastication, frequently lasting for several hours. The food may be regurgitated after each meal, and also at intervals during the day, while some persons with the habit will rechew the food only at special times. The condition seems confined mostly to the male sex, and exerts but little influence upon the general health, although, as a natural consequence, if the habit is long continued, the stomach is bound to suffer, and if the regurgitated food is not properly masticated gastric catarrh may result. The health mostly continues good as long as the food is properly chewed and the taste remains pleasant. As soon as the taste becomes sour or acid, it becomes evident that morbid alterations are taking place in the stomach, the health then suffering.

Treatment consists in removing the cause as far as possible, and minute attention to the food and manner of eating. The patient should be directed to eat slowly and properly masticate. Solid food, for a time at least, should be prohibited, or, if used, should be taken in small amounts. An effort should be made to repress the movements which cause the regurgitation, and the dorsal or other position unfavorable to the act should be insisted on for a definite time after each meal. Gastric sedatives, such as small pieces of ice swallowed at intervals, are useful, while tonics and laxatives are of service in some cases. Strychnine and electricity should be given a thorough trial, the latter being used over the region of the stomach or internally directed to the cardiac orifice.—*Medical Record.*

